# A Case of Asymptomatic Pulmonary Artery Aneurysm with Review of Management Strategies

**DOI:** 10.1155/2020/8885260

**Published:** 2020-10-14

**Authors:** Muhammad Talha Ayub, Allison Zimmerman, Wajeeha Rasool, Dinesh Kalra, Melissa Tracy, Anupama K. Rao

**Affiliations:** ^1^Division of Cardiovascular Medicine, RUSH University Medical Center, Chicago IL, USA; ^2^Department of Medicine, RUSH University Medical Center, Chicago IL, USA; ^3^Department of Medicine, Amita Health Presence St Francis Hospital, Evanston IL, USA

## Abstract

Pulmonary artery aneurysm (PAA) is defined as pulmonary artery diameter greater than 4 cm. With advances in cardiac imaging, the detection rate has increased but the natural history remains unknown. We present the case of a large, incidentally identified PAA in a patient with a history of congenital pulmonic stenosis.

## 1. History of Presentation

A 57-year-old Caucasian female presented to the cardiology clinic for evaluation of an incidentally diagnosed pulmonary artery aneurysm that was previously identified during workup of epigastric pain. Her epigastric pain was responsive to a trial of proton pump inhibitors. The patient was asymptomatic when she presented to the clinic. Vital signs on presentation were remarkable for blood pressure of 118/70 mmHg, temperature 98.2 F, heart rate 66/min, and respiratory rate 16/min. Her cardiopulmonary physical exam was unremarkable, with a normal S1 and S2 and without an audible murmur.

## 2. Past Medical History

- Moderate to severe congenital pulmonic stenosis (PS) diagnosed at 2 years of age based on right heart catheterization (RHC) findings; right ventricular (RV) systolic pressure 70 mmHg, mean pulmonary artery (PA) pressure 30 mmHg, and transvalvular systolic pressure gradient of 50 mmHg. Repeat RHC at the age of 5 years showed spontaneous regression to mild pulmonic stenosis; RV pressure of 50 mmHg, mean PA pressure 16 mmHg, with a transvalvular systolic pressure gradient of 30 mmHg
- Idiopathic ventricular tachycardia status post right ventricular outflow tract (RVOT) ablation at age 52

## 3. Differential Diagnosis and Etiology of PAA

- Congenital structural cardiac anomalies that alter hemodynamics and increase stress on the pulmonary artery: pulmonary valve stenosis, persistent ductus arteriosus, ventricular septal defects, atrial septal defects, hypoplastic aortic valve, and bicuspid aortic valve
- Pulmonary hypertension
- Trauma (iatrogenic from previous right heart catheterization)
- Infections: tuberculosis and syphilis
- Connective tissue diseases: Marfan syndrome, Ehlers-Danlos syndrome, and cystic medial necrosis
- Vasculitides: Bechet disease and giant cell arteritis

## 4. Investigations

**Figure 1:**
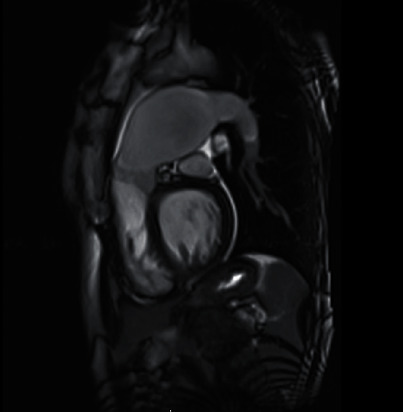
Cardiac MR RVOT view showing large PAA.

**Figure 2:**
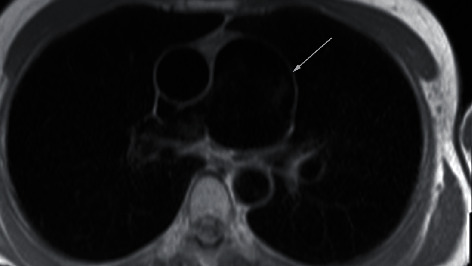
Cardiac MR axial localizer image showing PAA.

Routine laboratory testing did not reveal any abnormalities. Electrocardiogram was revealing of normal sinus rhythm with left bundle branch block morphology of QRS complex. Transthoracic echocardiogram was remarkable for normal left ventricular systolic function. Right atrium and ventricle were mildly dilated with normal systolic function. Pulmonic valve was not visualized. Cardiac MRI performed, at our facility, prior to her clinic visit demonstrated a large 58 × 57 mm aneurysm of the PA and mild pulmonic valve thickening (Figures 1 and 2) with mild PS by velocity encoded phase contrast imaging (peak velocity 1.6 m/s and peak gradient 10 mmHg).

## 5. Management

**Figure 3:**
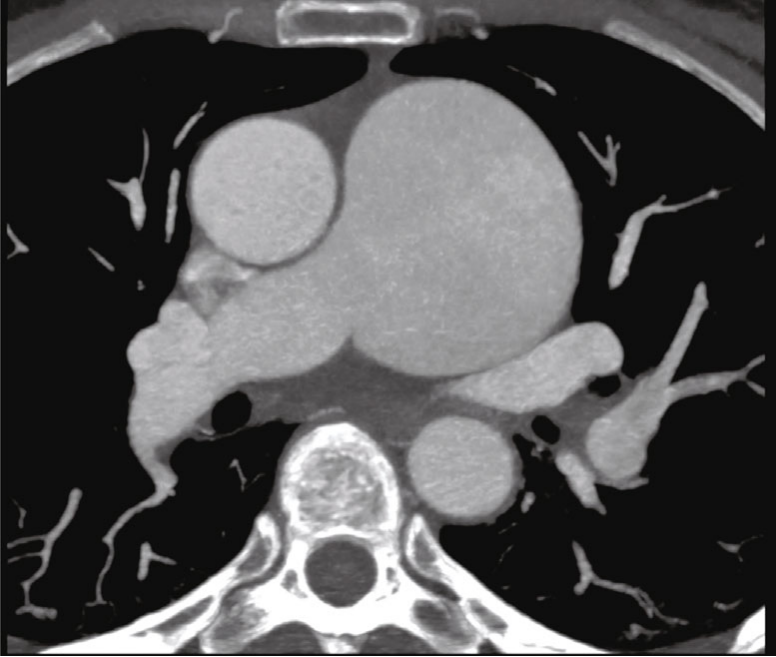
Cardiac CT axial images showing a 6.2 × 6.1 cm PAA.

**Figure 4:**
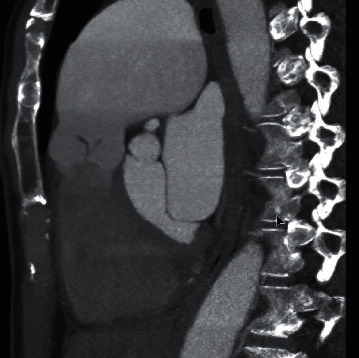
Cardiac CT sagittal section with PAA and pulmonic valve anatomy.

**Figure 5:**
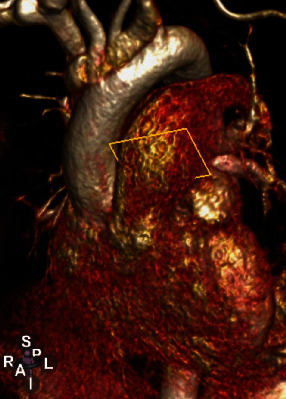
Cardiac CT 3D reconstruction.

Given the large size of the PAA, the patient was evaluated by an adult congenital heart specialist as well as by the cardiovascular surgery service. She underwent right and left heart catheterization that demonstrated a normal peak gradient (8 mmHg) across the pulmonic valve, normal right- and left-sided pressures, and angiographically normal coronary arteries. Cardiac CT was performed for additional anatomic assessment with a PAA of 62.6 × 61.7 mm without thrombus within the aneurysm sac (Figures 3, 4 and 5). The pulmonic root and RVOT were normal in size; there was no evidence of compression of adjacent structures (Figures 3 and 5). Surgical intervention was ultimately deferred due to her asymptomatic status and absence of high-risk features such as pulmonary hypertension, sac thrombus, compressive effect on the surrounding structures, and insufficient evidence for rate of growth of the aneurysm. Multidisciplinary cardiovascular team discussion concluded medical management with close follow-up was the best strategy. The patient is scheduled for serial imaging to monitor for growth in the size of the aneurysm and clinical surveillance for development of resultant complications. She will undergo annual transthoracic echocardiogram to monitor for development of pulmonary regurgitation and pulmonary hypertension. Strict blood pressure control was also recommended. She was instructed to avoid heavy lifting and high intensity workouts.

## 6. Discussion

Our patient had a large PAA presumably due to poststenotic dilatation from congenital, self-resolving, moderate to severe PS. Pulmonic stenosis has frequently been described as an isolated cause of PAA formation (1). PAAs were believed to be exceedingly rare but are increasingly recognized in the era of modern imaging. In a review of 109,571 autopsy cases over 100 years, only 8 cases of PAAs were identified (incidence rate 0.0073%) (2). Currently, there is no consensus regarding treatment of PAA based on size criteria; additionally, there are no clear guidelines delineating the roles of medical or surgical/percutaneous therapies for PAA. Treatment strategies include conservative management, endovascular repair, and surgical repair via aneurysmorrhaphy or PA replacement with allogeneic/synthetic grafts.

Conservative management is reasonable in the absence of pH, PAA thrombus, compression of adjacent structures, ≥5 mm increase in PAA diameter in 6 months, absolute PA diameter>5.5 cm, evidence of valvular pathology or shunt flow, and/or signs of rupture/dissection. Conservative management is focused on the treatment of underlying cause, especially in cases of infectious and vasculitis mediated etiologies. Favorable hemodynamic effects of beta blockers and diuretics (which decrease PA pressure and shear stress on the vessel wall) appear to be key features in the medical management of idiopathic PAAs (3).

While the size of the PAA (>5.5 cm) in this case could be considered large enough to necessitate surgical repair, the patient was asymptomatic and had evidence of low pressures within the PA system, with absence of high risk features on imaging. A conservative approach was deemed reasonable after a detailed multidisciplinary team and patient discussion considering the high risk to benefit ratio for surgical correction. Elevated PA pressure, due to medial necrosis, is by far the most important determinant favoring surgical intervention (4). A systematic review by Duijnhouwer et al. showed that the presence and duration of elevated intravascular pressure is the most important predictive factor for complications in PAA associated with congenital diseases (5). In patients who have low pressure systems, as in our patient, there appears to be a low incidence of PAA dissection (6).

Reisenauer et al. have recommended a management strategy by classifying patients into three groups. Those with “giant” aneurysm (greater than 8 cm) should undergo surgical intervention. PAAs between 5 and 8 cm are candidates for surgical intervention if symptomatic have evidence of serial growth or a high-pressure PA system. Those with PAAs less than 5 cm should be monitored with serial imaging (4).

In cases where intervention is needed, there are several surgical approaches described in the literature. Aneurysmorrhaphy is recommended in patients with an area of focal dilatation of the pulmonary artery. It involves excision and repair of the most dilated part of the aneurysm. Although there are no specific guidelines for endovascular vs. surgical repair of PAA, most of the literature suggests that endovascular treatment be offered when feasible as there is less morbidity and mortality. Other endovascular options that have been described include the use of coil embolization, vascular plugs, stent-assisted coil embolization, and stent grafting of the pulmonary arteries (7) {Park, 2018 #254}. While endovascular treatments carry less risk compared to surgery, they maintain similar risks to other endovascular embolization procedures elsewhere in the body including nontarget embolization, arterial thrombosis, arterial dissection, contrast-induced nephropathy, and end-organ damage. More invasive options involve complete replacement of the entire pulmonary trunk with a synthetic graft. This approach is typically used for PAAs secondary to infectious and inflammatory diseases (3). In cases where the pulmonic valve is involved, repair or replacement should be considered for the relief of right ventricular volume overload and vessel wall burden. In fact, aneurysmorrhaphy alone without pulmonic valve replacement was associated with recurrence of PA dilatation, possibly as a result of hemodynamic stress on the vessel wall (8). Treatment of distal PAAs is more complex and may require lung resection and combined heart-lung transplantation, particularly in patients with PAH (1). There is a lack of data regarding morbidity and mortality in relation to surgery, because no large series of PAA patients have been studied; however, it has been suggested that perioperative morbidity is comparable to that of aortic aneurysm repair (1). Given the lack of consensus on guidelines for treatment, a multidisciplinary approach between specialists is necessary to increase survival and minimize the risk of procedure-related complications.

## 7. Conclusions

1. PAAs are a rare albeit important clinical entity with an increase in the incidence due to the advent of multimodality cardiac imaging
2. Careful management is crucial because of the fatal complications including rupture, dissection, pulmonary embolism, and heart failure
3. An absolute diameter threshold for intervention does not exist. We believe that there should be a higher threshold for surgical management of PAA than for aneurysms of the aorta, especially when the PA pressure is normal
4. Pulmonary artery pressure, aneurysm size, symptoms, and etiology of PAA are the important determinants of treatment strategy

### 7.1. Take-Home Messages/Learning Objectives

1. Definite thresholds for surgical correction of PAA do not exist. Studies aiming at describing the natural history of PAAs, risk of complications, and optimal management strategy are needed
2. Patients with PAAs should be closely monitored for signs and symptoms of heart failure, pulmonary insufficiency, pulmonary hypertension, and/or rapid increase in the size in which case surgical correction should not be delayed

## Data Availability

Previously reported cases and reviews were used to support this study and are available online at Pubmed. These prior studies are cited at relevant places within the text as references.
